# THE EFFECT OF SOCIAL SUPPORT ON QUALITY OF LIFE OF PERSONS WITH COGNITIVE IMPAIRMENT

**DOI:** 10.1093/geroni/igz038.3308

**Published:** 2019-11-08

**Authors:** Wilma E Afunugo, Rafael Samper-Ternent

**Affiliations:** 1 University of Texas Medical Branch at Galveston, Galveston, Texas, United States; 2 University of Texas Medical Branch, Galveston, Texas, United States

## Abstract

According to the Alzheimer’s Association, 5.6 million Americans age 65 and older are living with Alzheimer’s Disease. Since pharmacological treatments have yet to be developed, we want to determine whether the amount and quality of social support influence the quality of life (QoL) of persons with dementia so they can lead active and purposeful lives. We analyzed data from 22,030 individuals aged 50+ from the 2010 Health and Retirement Study cohort. The dependent variable, QoL, was measured as self-rated health. The main independent variable, cognitive status, was obtained through direct and proxy interview measurements of cognition. For social support, a composite score including the number of social contacts/close relationships and perceived social support/strain was created. Lastly, several covariates were included. Longitudinally, we examined how QoL changed between 2010 and 2012 using 3 stepwise regression models. Model 1 found those with normal cognition have lower odds of poor QoL vs. those with cognitive impairment (OR = 0.38, p <.0001), number of relationships and perceived social support decreases the odds of poor QoL (p = 0.003, p <.0001), while social strain increases the odds of poor QoL (p <.0001). Model 3 revealed similar findings but also, persons with comorbidities have increased odds of poor QoL (p <.0001), while persons with better function have decreased odds of poor QoL (p <.0001). In conclusion, these results can be used to design interventions to improve social support and reduce social strain, which can also improve QoL for dementia caregivers.

